# Chemical Constituents of the Leaves of *Diospyros kaki* (Persimmon)

**DOI:** 10.3390/plants10102032

**Published:** 2021-09-28

**Authors:** Jaeyoung Kwon, Jeong-Eun Park, Jin-Su Lee, Jung-Hwan Lee, Hoseong Hwang, Sang-Hoon Jung, Hak-Cheol Kwon, Dae-Sik Jang

**Affiliations:** 1 KIST Gangneung Institute of Natural Products, Korea Institute of Science and Technology (KIST), Gangneung 25451, Korea; kjy1207@kist.re.kr (J.K.); 217513@kist.re.kr (J.-H.L.); hoseong91@kist.re.kr (H.H.); shjung@kist.re.kr (S.-H.J.); 2 Department of Life and Nanopharmaceutical Sciences, Graduate School, Kyung Hee University, Seoul 02447, Korea; 530pje@naver.com (J.-E.P.); lee2649318@naver.com (J.-S.L.)

**Keywords:** *Diospyros kaki*, persimmon leaves, flavonoid, antioxidant

## Abstract

*Diospyros kaki* (persimmon) leaves have long been utilized as traditional medicine for the treatment of ischemic stroke, angina, and hypertension and as a healthy beverage and cosmetic for anti-aging. This study aimed to isolate as many compounds as possible from an ethanol extract of the persimmon leaves to identify the biologically active compounds. The antioxidative effect of the ethyl acetate layer from the ethanol extract of the persimmon leaves was demonstrated by 2,2-diphenyl-1-picrylhydrazyl (DPPH) assay and online high-performance liquid chromatography-2,2′-azino-bis(3-ethylbenzothiazoline-6-sulfonic acid) (HPLC-ABTS) analysis. A new flavonoid, kaempferol-3-*O*-*β*-d-2″-coumaroylgalactoside (**1**), and a new natural compound, kaempferol-3-*O*-*β*-d-2″-feruloylglucoside (**3**) were isolated from the ethyl acetate layer, along with 25 previously known compounds, including fourteen flavonoids, one ionone, two coumarins, seven triterpenoids, and one acetophenone. Their structures were determined by the interpretation of spectrometric and spectroscopic data. All isolated compounds were rapidly evaluated using an online HPLC-ABTS assay, and of these, compounds **4**–**8**, **11**, **13**, **15**, and **16** clearly showed antioxidative effects. The amount of these compounds was 0.3–0.65% of the extract.

## 1. Introduction

*Diospyros kaki* Thunb. (persimmon) belongs to the family of Ebenaceae and is widely distributed in Korea, China, and Japan. Its fruit is eaten fresh or dry, while the leaves have long been used as a traditional medicine to treat ischemic stroke, angina, hypertension, atherosclerosis, and infectious diseases [1]. Furthermore, its leaves have been utilized as healthy beverages and cosmetics due to their anti-aging properties and abilities to help prevent cholesterol and melanin accumulation [1]. Recent research has suggested that the extracts of the persimmon leaves possess a wide range of biological properties, including radical scavenging, neuroprotection, thrombosis inhibition, anti-atherosclerosis, and anti-allergy [2,3,4,5,6]. A previous phytochemical investigation suggested that various types of flavonoids and terpenoids are the main constituents [7], and several tannins, naphthoquinones, coumarins, ionones, and fatty acids were also reported [8,9,10,11,12].

Reactive oxygen species (ROS) are reactive molecules produced in biological systems, and the balance between the generation and elimination of ROS is well controlled in normal cellular physiology [13]. However, excessive generation of ROS causes oxidative damage, and in turn, aging and age-related diseases including cancer, diabetes, and Parkinson’s disease [14]. Hence, discovering antioxidants such as flavonoids and phenolic compounds could be a promising strategy to treat these diseases.

As part of our continuous project to find biologically active compounds [15], the antioxidative effect of the ethanol (EtOH) extract and solvent partitions from the persimmon leaves was evaluated using 2,2-diphenyl-1-picrylhydrazyl (DPPH) assay and online high-performance liquid chromatography-2,2′-azino-bis(3-ethylbenzothiazoline-6-sulfonic acid) (HPLC-ABTS) analysis. A phytochemical study on the persimmon leaves led to the isolation of one new flavonoid (**1**) and one new natural compound (**3**), along with 25 previously known compounds. The structures were characterized by the application of spectroscopic and spectrometric methods. All isolated compounds were rapidly screened for their antioxidative effects using online HPLC-ABTS. Furthermore, the quantitative analysis of all isolated compounds was performed in the present study.

## 2. Results and Discussion

### 2.1. Antioxidative Effect of the Persimmon Leaves

**Figure 1:**
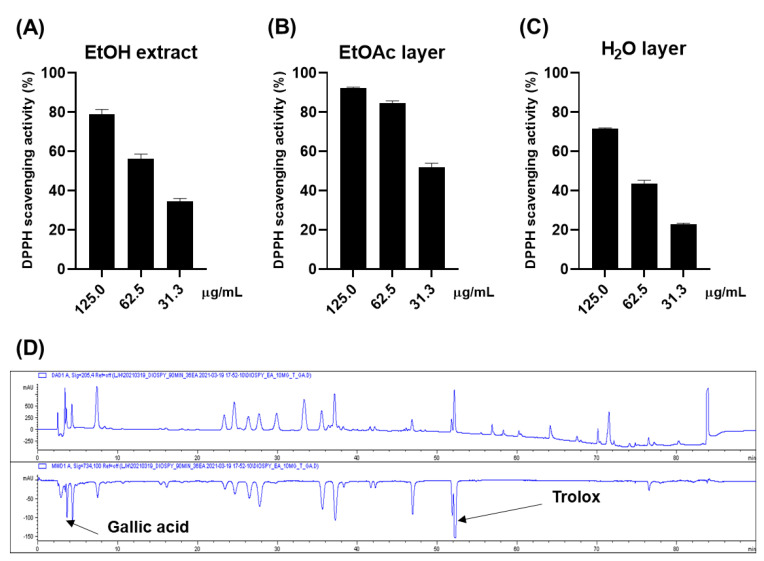
DPPH scavenging effects of the EtOH extract (**A**), EtOAc layer (**B**), and H_2_O layer (**C**) H_2_O layer of the persimmon leaves; (**D**) online ABTS-HPLC chromatogram of the EtOH extract.

The antioxidative effect of the ethanol (EtOH) extract of persimmon leaves was evaluated for a preliminary screening through DPPH (Figure 1A). The 0.125 mg/mL of the extract scavenged approximately 80% of the DPPH radical, while 0.025 mg/mL of ascorbic acid made up 94% of the radical. The online HPLC-ABTS assay was carried out to rapidly ensure the reliability of these results (Figure 1D). Gallic acid and Trolox were used as internal standards. The chromatogram at 734 nm (negative peak) suggested that approximately nine constituents could have antioxidative activities. Gallic acid and Trolox (6-hydroxy-2,5,7,8-tetramethylchroman-2-carboxylic acid) were used as internal standards to ensure the reliability of the results. It was inferred that most of these peaks were flavonoid derivatives such as flavonoid glycoside and flavanol, based on the dereplication analysis performed by comparing ultraviolet (UV) and mass spectra of the compounds with the published data. Bioassay-guided fractionation suggested that these antioxidative compounds were abundant in the ethyl acetate (EtOAc) layer, while the water (H_2_O) layer showed weak activity (Figure 1B,C).

### 2.2. Phytochemical Investigation

**Figure 2:**
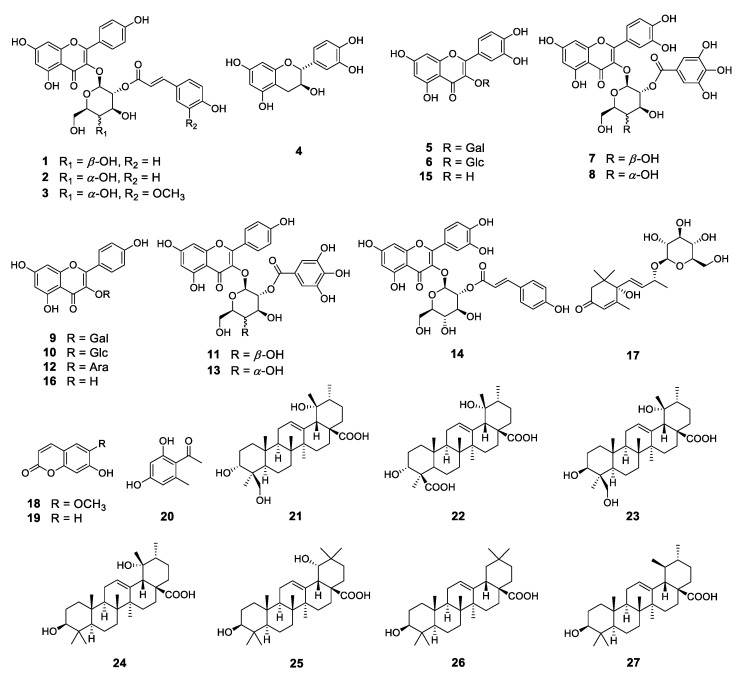
Structures of the compounds isolated from *D. kaki* leaves.

**Figure 3:**
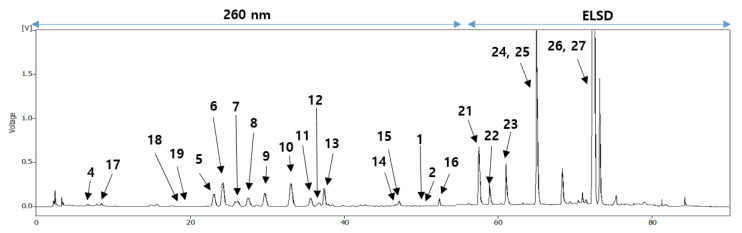
HPLC chromatogram of the EtOH extract from the persimmon leaves.

To identify these antioxidative compounds, various chromatographic and spectroscopic methods were carried out for the isolation and structural characterization of the compounds. A new flavonoid (**1**) and a new natural compound (**3**) were obtained from the ethyl acetate layer of the ethanol extract, together with 25 previously reported compounds, namely kaempferol-3-*O*-*β*-2″-coumaroylglucoside (**2**) [16], (+)-catechin (**4**) [17], hyperoside (**5**) [17], isoquercitrin (**6**) [18], quercetin-3-*O*-*β*-2″-galloylgalactoside (**7**) [19], quercetin-3-*O*-*β*-2″-galloylglucoside (**8**) [20], trifolin (**9**) [18], astragalin (**10**) [18], kaempferol-3-*O*-*β*-2″-galloylgalactoside (**11**) [21], kaempferol-3-*O*-*α*-arabinoside (**12**) [22], kaempferol-3-*O*-*β*-2″-galloylglucoside (**13**) [23], quercetin-3-*O*-*β*-2″-coumaroylglucoside (**14**) [24], quercetin (**15)** [15], kaempferol (**16**) [25], (6*S*,9*S*)-roseoside (**17**) [26], scopoletin (**18**) [27], umbelliferone (**19**) [28], 1-(2,4-dihydroxy-6-methylphenyl)ethanone (**20**) [29], barbinervic acid (**21**) [30], diospyric acid B (**22**) [7], rotungenic acid (**23**) [31], pomolic acid (**24**) [32], siaresinolic acid (**25**) [33], oleanolic acid (**26**) [25], and ursolic acid (**27**) [25] by using spectroscopic and spectrometric and physical data in comparison with the published data and also with thin layer chromatography (TLC) analysis (Figure 2 and Figure 3). Among these, compounds **2**, **16**, **17**, **19**, **20**, and **24** were firstly isolated from *D. kaki*.

Compound **1** was obtained as a yellow powder, in which the molecular formula was established as C_30_H_26_O_13_ based on high-resolution mass spectrometry (HRMS) data. The UV spectrum exhibited absorption bands at 207 and 315 nm, indicating that compound **1** had a flavonol backbone. The ^1^H nuclear magnetic resonance (NMR) data (Table 1, Figure S1) showed a typical pattern of coumaroylated flavonol glycoside, showing two sets of AA′BB′-type signals (*δ*_H_ 8.00 (2H, d, *J* = 8.5 Hz, H-2′ and H-6′), 6.87 (2H, d, *J* = 8.5 Hz, H-3′ and H-5′)) in the B ring of kaempferol as well as the signals (*δ*_H_ 7.45 (2H, d, *J* = 8.5 Hz, H-2′′′ and H-6′′′), 6.81 (2H, d, *J* = 8.5 Hz, H-3′′′ and H-5′′′) in the aromatic ring of the coumaroyl group. Two doublet signals (*δ*_H_ 7.65 (1H, d, *J* = 15.5 Hz, H-7′′′) and *δ*_H_ 6.35 (1H, d, *J* = 16.0 Hz, H-8′′′) were observed, indicating trans-olefinic protons of the coumaroyl group. Additionaly, an anomeric proton signal (*δ*_H_ 5.57 (1H, d, *J* = 8.0 Hz, H-1′′) was observed in the sugar region, suggesting the presence of the *β*-configurated cyclic sugar group. The ^13^C and distortionless enhancement by polarization transfer NMR data (Table 1, Figure S2) showed 30 resonances comprising two trans-olefinic carbons, ten aromatic carbons, and six glucosyl moiety carbons, and ten non-protonated carbons including two carbonyl carbons. In particular, the chemical shifts at C-2, C-3, and C-4 (*δ*_C_ 158.1, 134.9, and 179.2) were characteristic signals of flavonol 3-*O*-glycoside. Additionally, the carbonyl carbon signal at C-1′′′ (*δ*_C_ 168.7) and two trans-olefinic carbon signals at C-2′′′ and C-3′′′ (*δ*_C_ 146.9, 115.2) were typical chemical shifts of the coumaroyl group. The anomeric carbon signal at C-1′′(*δ*_C_ 100.4) and other signals for the glycosyl moiety from C-2′′ to C-6′′ (*δ*_C_ 74.3, 73.4, 70.5, 77.4, and 62.0) were observed. These one-dimensional (1D) NMR data were superimposable to those of kaempferol-3-*O*-*β*-2′′-coumaroylglucoside (**2**) [16]. However, the careful comparison of the ^13^C NMR data between the two compounds suggested that compound **1** had a galactose moiety, which was further demonstrated by the nuclear Overhauser enhancement spectroscopy (NOESY) NMR data (Figure S6). While the NOESY correlation between H-2′′ and H-4′′ was observed in compound **2**, there was no correlation between these protons in compound **1**. In general, interpreting ^13^C NMR and NOESY NMR data is an effective method to determine the type of glycosyl moiety. The location of the galactose moiety was deduced to be at C-3 according to the downfield shift of C-2 and C-4, as further evidenced by the heteronuclear multiple bond correlation (HMBC) between H-1′′ and C-3 (Figure S5). The position of the coumaroyl group was demonstrated to be at C-2′′ based on the downfield shift (*δ*_H_ 5.36 (1H, dd, *J* = 10.0, 8.0 Hz, H-2′′)) and the HMBC correlation between H-2′′ and C-1′′′. As a result, the structure of compound **1** was determined as kaempferol-3-*O*-*β*-2″-coumaroylgalactoside. Although compound **2** was previously isolated from various sources, including *Quercus suber* [16] and *Allium porrum* [34], compound **1** was isolated and structurally characterized for the first time.

Compound **3** was isolated as a yellow powder, and the molecular formula was established as C_31_H_28_O_14_ by analyzing HRMS data. The UV spectrum showed the UV absorption at 210 and 327 nm due to the same aglycone with compounds **1** and **2**. The ^1^H NMR data (Table 1, Figure S10) were similar to those of compound **2**, but compound **3** had a feruloyl group instead of the coumaroyl group, as evidenced by the presence of an additional methoxy group (*δ*_H_ 3.91 (3H, s, 3′′′-OCH_3_)). Additionally, an anomeric proton signal (*δ*_H_ 5.64 (1H, d, *J* = 8.0 Hz, H-1′′)) was observed, indicating that the glycosyl linkage was a *β*-configuration, and the downfield-shifted signal (5.03 (1H, t, *J* = 8.5 Hz, H-2′′)) was shown in the sugar region, as with compound **1**. The ^13^C NMR data (Table 1, Figure S11) showed 31 resonances comprising two *trans*-olefinic carbons, ten quarternary carbons, ten aromatic carbons, six glucosyl moiety carbons, and one methoxy carbon, and ten non-protonated carbons, including two carbonyl carbons corresponding to kaempferol, feruloyl, and glucose groups. In particular, carbon signals from C-2′′ to C-6′′ (*δ*_C_ 75.8, 76.3, 71.5, 78.8 and 62.5) suggested the presence of a glucose moiety. The locations of the glucose moiety and feruloyl group were assigned by the long-range HMBC correlations between H-1′′ and C-3 (*δ*_C_ 134.8) and H-2′′ and C-1′′′ (*δ*_C_ 168.4) (Figure S14). The position of an additional methoxy group was determined by the key correlation between 3′′′-OCH_3_ and C-3′′′ (*δ*_C_ 149.4). The above results suggested the structure of compound **3** as kaempferol-3-*O*-*β*-2”-feruloylglucoside. To the best of our knowledge, compound **3** was only reported as a product of the hydrolysis of 3-*O*-*β*-(2-*O*-feruloyl)-glucosyl-7,4′-di-*O*-*β*-glucosylkaempferol, isolated from *Allium tuberosum* [35]. Therefore, the structure of **3** was elucidated as a new natural compound.

Compound **11** was isolated as a yellow powder. The ^1^H NMR data (Figure S19) displayed a set of AA′BB′-type signals (*δ*_H_ 8.06 (2H, d, *J* = 9.0 Hz, H-2′, H-6′), 6.87 (2H, d, *J* = 9.0 Hz, H-3′, H-5′)) in the B ring of kaempferol and a singlet signal at *δ*_H_ 7.02 (2H, s, H-3′′′, H-7′′′) of a galloyl moiety in aromatic region, which is a characteristic signal of galloylated flavonol. An anomeric proton signal (*δ*_H_ 5.78 (1H, d, *J* = 8.0 Hz, H-1′′)) indicated that the glycosyl linkage was a *β*-configuration. Furthermore, a downfield shifted proton signal (5.27 (1H, t, *J* = 9.5 Hz, H-2′′)) suggested that the galloyl group was attached at the hydroxyl group of C-2′′ because this shift could be attributed to the anisotropic influence of the *O*-galloyl moiety [21]. The ^13^C NMR data (Figure S20) exhibited 26 resonances, indicating galloylated flavonol glycoside. The carbon signals from C-2′′ to C-6′′ (*δ*_C_ 71.1, 72.7, 68.2, 76.0, and 60.1) suggested the presence of a galactose moiety. Therefore, the structure of compound **11** was confirmed as kaempferol-3-*O*-*β*-2′′-galloylgalactoside. Although compound **11** was previously isolated from various sources, including *D. kaki* [21,36], only the ^1^H NMR and MS data were previously reported. Thus, the ^13^C NMR data was reported for the first time in this study.

### 2.3. Antioxidative Activities of the Isolated Compounds

**Figure 4:**
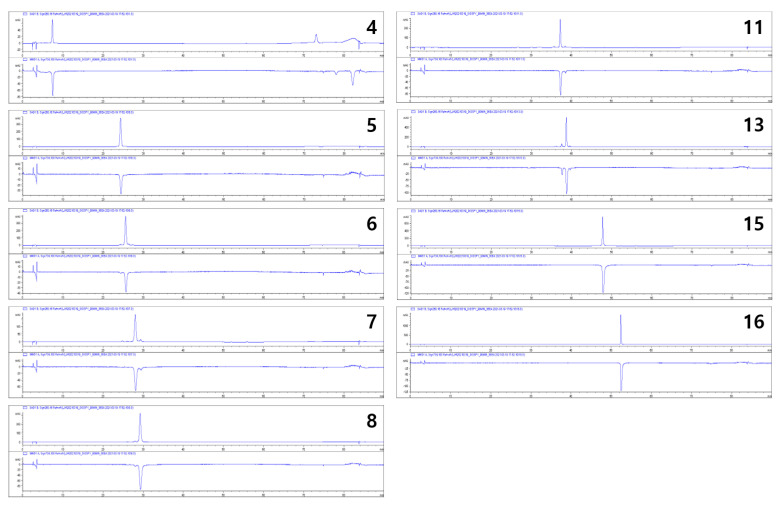
Radical scavenging activities of the isolated compounds (**4**–**8**, **11**, **13**, **15**, and **16**) assessed by online HPLC-ABTS assay.

All isolated compounds were evaluated for their antioxidative effects using rapid online HPLC-ABTS analysis to determine which compounds contributed to the antioxidative effect of the persimmon leaves. Compounds **4**–**8**, **11**, **13**, **15**, and **16** showed potent antioxidative activities (Figure 4). Most of these compounds were kaempferol and quercetin derivatives, but some derivatives (**1**–**3**, **9**, **10**, **12**, and **14**) did not show activities. The structure-activity relationship was not fully determined but was partially revealed. In particular, quercetin and kaempferol with galloyl moieties (**7**, **8**, **11**, and **13**) were found to have potent activities, but those with coumaroyl or feruloyl moieties (**1**–**3** and **16**) did not show any activities.

### 2.4. Quantitative Analysis of Isolated Compounds

The quantitative analysis of all isolated compounds in the EtOAc-soluble extract was performed to confirm that the antioxidative effects of the persimmon leaves could be caused by these active compounds. A specific HPLC method with diode array detection and evaporative light scattering detector was developed for the simultaneous determination of 27 compounds. The contents of all isolated triterpenoids were approximately 5.9% of the extract, and of these, those of siaresinolic acid (**25**), oleanolic acid (**26**), and ursolic acid (**27**) accounted for a significant portion, as reported in previous studies [7]. The contents of flavonoids were approximately 5.4%, and of these, those of active compounds were 3.2% (Table 2). In particular, the contents of kaempferol-3-*O*-*β*-2′′-galloylgalactoside (**9**), kaempferol-3-*O*-*β*-2″-galloylglucoside (**10**), isoquercitrin (**13**), and quercetin-3-*O*-*β*-2″-galloylglucoside (**15**) were more than 0.3%. The analytical method was verified using a simple validation procedure to ensure the relevance of the method, which showed adequate specificity, linearity, accuracy, and precision.

## 3. Discussion

Phytochemical investigations to identify biologically active compounds in persimmon leaves have been widely carried out. So far, a considerable number of triterpenoids and flavonoids, including kaempferol and quercetin derivatives, have been reported from *D. kaki* [1]. In this study, we obtained 27 compounds, including sixteen flavonoids, one ionone, two coumarins, seven triterpenoids, and one acetophenone. Of these, compound **1** was found to be a new flavonoid and compound **2** was firstly isolated from *D. kaki*. Additionally, kaempferol-3-*O*-*β*-2′′-feruloylglucoside (**3**) was only reported as a hydrolyzed product of 3-*O*-*β*-(2-*O*-feruloyl)-glucosyl-7,4′-di-*O*-*β*-glucosylkaempferol (**3**), isolated from *Allium tuberosum* [35]. Compound **3** was not only obtained directly from a natural source for the first time but has also not been reported in *D. kaki* previously. Furthermore, kaempferol-3-*O*-*β*-2′′-galloylgalactoside (**11**) has been previously reported in many sources, including *D. kaki*, but only the ^1^H NMR and MS have been reported due to the lack of detailed research. Hence, the ^13^C NMR data was reported for the first time here.

Until now, there have been few studies that demonstrated the antioxidative abilities of extracts or fractions of persimmon leaves [37,38]. Most studies used rapid assay methods such as DPPH or ABTS assays. In particular, in the previous paper, 200 μg/mL of flavonoid-rich fraction exhibited 68.73% inhibition of DPPH radical. Aside from this result, however, this fraction also showed superoxide anion radical scavenging, hydroxyl radical scavenging, and metal chelating activities [38]. Although we did not evaluate these assays, bioassay-guided isolation was carried out because the ethanol extract and ethyl acetate fraction in the present study showed comparable DPPH radical scavenging activity. Additionally, despite previous results, only a few studies to identify biologically active compounds have been carried out. A few secoiridoids and lignans showed radical scavenging activities [39]. In the case of flavonoids, there have been several reports that quercetin, kaempferol, and their glycosides have antioxidative properties [40]. Antioxidative properties of galloylated kaempferol glycoside and galloylated quercetin glycoside obtained from other sources have been reported [41]. As yet, there have been no reports that each of these compounds derived from the persimmon leaves has antioxidative effects, except that a mixture of these compounds exhibited an antioxidative effect [21].

Additionally, so far, simultaneous determination of only a few triterpenoids or flavonoids has been carried out for the quantitative analysis of these compounds [42,43]. However, the present study suggests a method for simultaneous determination of most components in the persimmon leaves.

## 4. Materials and Methods

### 4.1. Plant Material

The leaves of *Diospyros kaki* Thunb. (Ebenaceae) were purchased at a domestic Korean herbal market in Yeongcheon in March 2018. The leaves were harvested at the basin area surrounded by mountains at an altitude of 800–1200 m in Gyeongsangbuk-do in August 2017. The average amount of the annual precipitation in this area was 1050 mm, of which, half fell between June and August. The average annual temperature was about 12.5 °C and the average relative humidity was 69%. The temperature at harvest time was approximately 37–40 °C. The obtained leaves were dried at approximately 45 °C in a plant dryer. A voucher specimen (DIKA1-2018) has been deposited in the Laboratory of Natural Product Medicine, College of Pharmacy, Kyung Hee University, Republic of Korea.

### 4.2. General Experimental Procedures

Melting points were obtained using MPA 100 (Stanford research systems, Sunnyvale, CA, USA) in open capillary tubes. Optical rotations were measured on a Jasco P-2000 polarimeter, using a 10 cm microcell. UV spectra were obtained on Optizen pop (Mecasys, Daejeon, Korea). HRMS data were obtained using a quadrupole time-of-flight (Q-TOF) micro mass spectrometer (Waters, Milford, MA, USA). NMR spectra were obtained using a JEOL 500 MHz spectrometer using tetramethylsilane as a reference signal, and chemical shifts are expressed as *δ* values. Infrared (IR) spectra were obtained using an Agilent Cary 630 FTIR (Agilent Technologies, Santa Clara, CA, USA). TLC analysis was performed on silica gel 60 F_254_ and RP-18 F_254S_ plates (Merck, Kenilworth, NJ, USA), and compounds were visualized by dipping plates into 20 % (*v*/*v*) H_2_SO_4_ reagent, which were then heated at 110 ℃ for 5-10 min. Column chromatography was performed using silica gel (70–230 or 230–400 mesh ASTM, Merck), Sephadex LH-20 (Amersham Pharmacia Biotech, Buckinghamshire, United Kingdom), Diaion HP-20 (Mitsubishi, Tokyo, Japan), and reversed-phase silica gel (ODS-A 12 nm S-150 μm, YMC, Tokyo, Japan). Flash chromatography was performed using a CombiFlash (Teledyne Isco, Lincoln, NE, USA) with pre-packed cartridges, RediSep-silica (12 g, 24 g, and 40 g) and RediSep-C18 (13 g, 26 g, 43 g, and 130 g). Preparative HPLC was performed using the Gilson purification system (Gilson, Middleton, WI, USA) with a YMC Pack ODS-A column (250 × 20.0 mm, 5.0 μm, YMC, Tokyo, Japan), a J’sphere ODS-M80 column (250 × 20.0 mm, 4.0 μm, YMC, Tokyo, Japan), and a Luna C18(2) column (250 × 21.2 mm, 10.0 μm, Phenomenex, Torrance, CA, USA). HPLC analysis was performed on a Youngin YL9100 HPLC system comprising an evaporative light scattering detector (Youngin, Anyang, Korea) with Luna C18(2) column (150 × 4.6 mm, 5.0 μm, Phenomenex, Torrance, CA, USA). The online HPLC-ABTS screening was performed on an Agilent 1200 HPLC system with a YMC Pack ODS-A column (150 × 4.6 mm, 5.0 μm). All solvents used for the chromatographic separations were distilled.

### 4.3. Extraction and Isolation

The dried plant material (15.0 kg) was extracted with 216 L of ethanol (EtOH) in a water bath at 60 °C for 4 h, and the solvent was evaporated to obtain EtOH extract (1.2 kg, yield 8%). The extract was suspended in H_2_O (2.1 L) and partitioned with ethyl acetate (EtOAc, 4.9 L × 3) to give EtOAc- (321.9 g, yield 2.15%) and H_2_O-soluble layers (748.0 g, yield 4.99%), respectively. The EtOAc-soluble layer (321.9 g) was subjected to a silica gel column (*ϕ* 10.5 × 35.0 cm) with *n*-hexane:EtOAc:methanol (MeOH) mixtures (from 8:1.8:0.2 to 0:0:1 *v*/*v*/*v*) to afford nine fractions (E1~E9).

Fraction E5 (14.2 g) was chromatographed over a Diaion HP-20 column (*ϕ* 5.0 × 29.0 cm) with acetone:H_2_O gradient (7:3 to 1:0) to afford seven fractions (E5-1~E5-7). Fraction E5-1 was subjected to a silica gel column with *n*-hexane:EtOAc:MeOH = 7:2.7:0.3 to 0:0:1 to afford 4 fractions (E5-1-1~E5-1-4). E5-1-1 (13.2 mg) and E5-1-2 (7.0 mg) were combined and purified by preparative (prep)-HPLC using an YMC Pack ODS-A column (H_2_O:MeOH = 27:23, 7 mL/min) to obtain compounds **19** (8.3 mg, t_R_ 26.0 min) and **20** (2.5 mg, t_R_ 24.0 min).

Fraction E8 (75.19 g) was fractionated into acetone-soluble (AS) and acetone-insoluble (AIS) fractions. Fraction AS (44.07 g) was chromatographed over a Diaion HP-20 column (*ϕ* 6.5 × 12.5 cm) with acetone:H_2_O mixtures (3:7 to 1:0) to afford 12 fractions (AS1~AS12). Fraction AS2 (2.5 g) was separated by a Sephadex LH-20 column (*ϕ* 4.7 × 51.0 cm) with MeOH to give nine fractions (AS2-1~AS2-9). Fraction AS2-2 (196.3 mg) was separated by flash chromatography (FC) using a RediSep-C18 cartridge (26 g, acetonitrile:H_2_O, 0:1 to 7:1) to yield compound **17** (28.6 mg). Fraction AS3 (3.1 g) was separated into 11 fractions using a Sephadex LH-20 column (*ϕ* 4.7 × 51.0 cm) with MeOH (AS3-1~AS3-11). Fraction AS3-6 (0.7 g) was separated by FC with RediSep-C18 (130 g, MeOH:H_2_O, 1:9 to 3:2) to give compounds **4** (51.8 mg), **5** (21.7 mg, t_R_ 42.5 min), and **6** (20.2 mg, t_R_ 47.0 min). Fraction AS4 (8.8 g) was subjected to a silica gel column (*ϕ* 5.2 × 21.0 cm) with MC:MeOH:H_2_O mixtures (from 8:1.8:0.2 to 7:2.7:0.3) to isolate Compounds **7** (5.0 mg), **8** (5.1 mg), **9** (20.0 mg), **10** (306.6 mg), and **12** (20.1 mg). Fraction AS5 was subjected to a silica gel column (*ϕ* 5.2 × 24.5 cm) with CH_2_Cl_2_:MeOH:H_2_O mixtures (8:1.8:0.2 to 7:2.7:0.3) to generate six fractions (AS5-1~ AS5-6) to afford compounds **11** (3.0 mg) and **13** (7.4 mg). Fraction AS10 was separated into seven fractions using a Sephadex LH-20 column (*ϕ* 3.5 × 50.5 cm) with MeOH (AS10-1~AS10-7). Fraction AS10-4 was separated by FC with a RediSep-C18 (43 g, MeOH:H_2_O, 0:1 to 3:2) cartridge to purify compounds **1** (5.3 mg), **2** (21.4 mg), **14** (15.9 mg), and **15** (40.4 mg). Fraction AS12 was separated into five fractions using a Sephadex LH-20 column (*ϕ* 3.5 × 50.5 cm) with MeOH (AS12-1~AS12-5). Compound **16** (20.1 mg) was obtained by recrystallization with MeOH from fraction AS12-5.

Fraction AIS (31.1 g) was chromatographed over a silica gel column with *n*-hexane:EtOAc:MeOH mixtures (8:1.8:0.2 to 0:0:1) as a mobile phase to afford 20 fractions (AIS1~AIS20). Fraction AIS5 was subjected to a silica gel column with *n*-hexane:EtOAc:MeOH mixtures (8:1.8:0.2 to 0:0:1) to afford compound **23** (224.7 mg). Fraction AIS6 was separated by FC with a RediSep-C18 (43 g, MeOH:H_2_O, 0:1 to 9:1) cartridge to give compound **25** (25.6 mg). Fraction AIS7 was subjected to a silica gel column with *n*-hexane:EtOAc:MeOH mixtures (8:1.8:0.2 to 0:0:1) to afford compounds **24** (100.0 mg) and **27** (214.1 mg). Fraction AIS10 was subjected to a silica gel column with *n*-hexane:EtOAc:MeOH mixtures (7:2.7:0.3 to 0:0:1) to isolate compounds **18** (5.0 mg) and **23** (16.7 mg). Fraction AIS11 was separated into 11 subfractions (AIS11-1~AIS11-11) by FC with a RediSep-C18 (130 g, MeOH:H_2_O, 1:1 to 4:1) cartridge. Compound **22** (37.8 mg) was obtained from fraction AIS11-4 by prep-HPLC with a J’sphere column. Fraction AIS12 was subjected to a silica gel column (*ϕ* 5.2 × 28.0 cm) with CH_2_Cl_2_:acetone mixtures (from 4:1 to 3:2) to afford compound **21** (188.0 mg). Finally, fraction AIS16 was separated using a Lichroprep RP-18 column (1.99 g, MeOH:H_2_O, 3:2 to 13:7) to obtain compound **3** (2.3 mg).

#### 4.3.1. kaempferol-3-*O*-β-d-2′′-coumaroylgalactoside (**1**)

Yellowish powder; m.p. 244.5 °C; [α]^22^_D_ -59.1° (*c* 0.1, MeOH); UV (MeOH) *λ*_max_ (log ε) 207 nm (3.98), 315 nm (3.92); IR (ATR) *ν*_max_ 3458, 2922, 1650, 1588, 1364, 1260, 1175, 1076, 834 cm^−1^; ^1^H and ^13^C NMR data, see Table 1; HRMS (positive mode) *m/z* 595.1447 [M + H]^+^ (calcd for C_30_H_27_O_13_, 595.1452).

#### 4.3.2. kaempferol-3-*O*-β-d-2′′-feruloylglucoside (**3**)

Yellowish powder; m.p. 225.2 °C; [α]^22^_D_ -119.6° (*c* 0.1, MeOH); UV (MeOH) *λ*_max_ (log ε) 210 nm (4.17), 327 nm (3.94); IR (ATR) *ν*_max_ 3369, 1652, 1599, 1512, 1360, 1264, 1177, 1076, 841 cm^−1^; ^1^H and ^13^C NMR data, see Table 1; HRMS (negative mode) *m/z* 623.1375 [M − H]^−^ (calcd for C_31_H_27_O_14_, 623.1401).

#### 4.3.3. kaempferol-3-*O*-β-d-2′′-galloylgalactoside (**11**)

Yellow powder; ^1^H NMR (500 MHz, DMSO-*d*_6_) *δ* ^1^H NMR 8.06 (2H, d, *J* = 9.0 Hz, H-2′, H-6′), 7.02 (2H, s, H-3′′′, H-7′′′), 6.87 (2H, d, *J* = 9.5 Hz, H-3′, H-5′), 6.39 (1H, s, H-8), 6.16 (1H, s, H-6), 5.78 (1H, d, *J* = 8.0 Hz, H-1′′), 5.27 (1H, t, *J* = 9.5 Hz, H-2′′); ^13^C NMR (125 MHz, DMSO-*d*_6_) δ 177.1 (C-4), 165.4 (C-7), 165.4 (C-1′′′), 161.2 (C-5), 160.1 (C-4′), 156.3 (C-2), 156.3 (C-9), 145.5 (C-4′′′), 145.5 (C-6′′′), 138.4 (C-5′′′), 132.5 (C-3), 131.0 (C-2′), 131.0 (C-6′), 120.7 (C-1′), 119.8 (C-2′′′), 115.2 (C-3′), 115.2 (C-5′), 108.9 (C-3′′′), 108.9 (C-7′′′), 103.8 (C-10), 98.8 (C-6), 98.8 (C-1′′), 93.7 (C-8), 76.0 (C-5′′), 72.7 (C-3′′), 71.1 (C-2′′), 68.2 (C-4′′), 60.1 (C-6′′).

### 4.4. Quantitative Analysis of Nine Compounds in the Persimmon Leaves

HPLC analysis was performed on a Waters HPLC system comprising 1525 pump and 2996 photodiode array detector (Waters, Milford, MA, USA). The UV wavelength was set at 260 nm. A Phenomenex Luna C18(2) column (150 × 4.6 mm, 5.0 μm, Phenomenex, Torrance, CA, USA) was used, and the injection volume was 10 μL. The column temperature was set at 25 °C. The mobile phase consisted of 0.02% trifluoroacetic acid (TFA, Sigma-Aldrich, St. Louis, MO, USA) in water (A) and acetonitrile (B) with a flow rate of 0.7 mL/min. The gradient conditions were as follows: 0–30 min, 15–20% B; 30–45 min, 20–35% B; 45–70 min, 35–100%; 70–80 min, 100%. The EtOH extract (10 mg) was dissolved in 10 ug/mL internal standard solution (1 mL). Simple method validation was carried out to ensure the relevance of the developed method and qualitative results. Five different solutions of each compound were analyzed to make each calibration curve. The intra- and inter-day precision and accuracy were confirmed through three replicates within a single day and three consecutive days. All samples were filtered through 0.2 μm membrane filters.

### 4.5. DPPH and Online HPLC-ABTS Analysis

The ability of samples to scavenge DPPH radicals was assessed based on a previous paper. Briefly, DPPH (0.1 mM) in methanol (100 μL) was mixed with various concentrations of samples (100 μL) for 1 h in the dark. The absorbance was recorded at 517 nm.

The online HPLC-ABTS analysis was performed based on the previous report with modifications. A mixed solution containing ABTS (0.08 mM) with potassium persulfate (0.12 mM) was made into an ABTS reagent. The reagent was stored at 4 °C for 12 h to stabilize radicals. All samples were analyzed by an Agilent HPLC system. The gradient conditions were the same as those used for quantitative analysis. The eluate was sent to a T-junction and reacted with ABTS reagent in a reaction coil at 40 °C. The chromatogram was visualized at 254 nm (positive peak), as well as at 734 nm (negative peak), to record the decrease in ABTS radicals.

## 5. Conclusions

In conclusion, this study presents a phytochemical investigation based on bioassay-guided isolation. As a result, a new flavonoid, kaempferol-3-*O*-*β*-d-2″-coumaroylgalactoside (**1**), and a new natural compound, kaempferol-3-*O*-*β*-d-2″-feruloylglucoside (**2**), were isolated, along with 25 previously known compounds, including fourteen flavonoids, one ionone, two coumarins, seven triterpenoids, and one acetophenone. All compounds were evaluated on antioxidative effects, and of these, nine flavonoids were found to possess activities. Simultaneous quantitative analysis was performed to confirm that the persimmon leaves have antioxidative effects due to these compounds.

## Figures and Tables

**Table 1:** ^1^H and ^13^C NMR data of compounds **1** and **3** in methanol-*d*_4_

Number of Carbon	1		3	
	*δ*_H_ Multi (*J* in Hz)	*δ*_C_	*δ*_H_ Multi (*J* in Hz)	*δ*_C_
2		158.1		158.6
		134.9		134.8
4		179.2		179.0
5		163.1		163.0
6	6.16 d (1.5)	101.2	6.11 d (2.0)	100.6
7		167.7		168.3
8	6.34 s	95.1	6.29 d (2.0)	95.2
9		158.5		158.1
10		105.3		105.2
1′		122.7		122.8
2′,6′	8.00 d (8.5)	132.1	7.98 d (9.0)	132.1
3′,5′	6.87 d (8.5)	116.3	6.88 d (9.0)	116.2
4′		161.6		161.6
1′′	5.57 d (8.0)	100.4	5.64 d (8.0)	100.7
2′′	5.36 dd (10.0, 8.0)	74.3	5.03 dd (9.0, 8.0)	75.8
3′′	3.75 dd (10.5, 3.5)	73.4	3.64 t (9.0)	76.3
4′′	3.89 d (3.5)	70.5	3.41 t (10.0)	71.5
5′′	3.55 t (6.0)	77.4	3.29 m	78.8
6′′	3.67 m	62.0	3.78 dd (12.0, 2.0)	62.5
			3.61 m	
1′′′		127.2		127.8
2′′′	7.45 d (8.5)	131.2	7.18 d (1.5)	111.7
3′′′	6.81 d (8.5)	116.8		149.4
4′′′		161.3		150.7
5′′′	6.81 d (8.5)	116.8	6.81 d (8.5)	116.5
6′′′	7.45 d (8.5)	131.2	7.07 dd (8.5, 1.5)	124.1
7′′′	7.65 d (15.5)	146.9	7.66 d (16.0)	147.2
8′′′	6.35 d (16.0)	115.2	6.37 d (16.0)	115.5
9′′′		168.7		168.4
3′′′-OCH_3_			3.91 s	56.4

**Table 2:** Quantitative analysis of nine compounds of the persimmon *leaves*.

No	Content (%)	Linear Range (μg/mL)	Regression Equation	*r* ^2^	Precision (% RSD)		Accuracy	
					Intraday	Interday	Intraday	Interday
4	0.33	31.25–500	y = 0.0014x + 0.0045	0.999	0.9 ± 0.2	0.9 ± 0.4	101.2 ± 0.3	102.3 ± 0.8
5	0.26	31.25–500	y = 0.0117x + 0.1248	0.999	1.1 ± 0.5	1.1 ± 0.7	101.3 ± 1.2	100.5 ± 1.5
6	0.65	31.25–500	y = 0.0118x + 0.1177	0.999	1.4 ± 0.7	1.1 ± 0.8	100.0 ± 2.5	99.4 ± 1.6
7	0.16	31.25–500	y = 0.0066x + 0.0548	0.998	1.9 ± 0.2	1.5 ± 0.7	101.2 ± 2.7	101.9 ± 0.2
8	0.48	31.25–500	y = 0.0113x + 0.0362	0.999	2.0 ± 1.5	1.3 ± 0.6	99.5 ± 1.4	101.3 ± 3.4
11	0.31	31.25–500	y = 0.0070x + 0.0433	0.998	1.1 ± 0.5	1.8 ± 0.8	100.3 ± 1.8	100.7 ± 1.0
13	0.58	31.25–500	y = 0.0097x + 0.1832	0.999	1.6 ± 0.3	1.3 ± 0.6	99.7 ± 1.8	100.2 ± 1.3
15	0.01	6.25–100	y = 0.0128x + 0.0076	0.999	1.6 ± 0.9	1.3 ± 0.8	102.9 ± 1.0	102.0 ± 1.0
16	0.38	6.25–100	y = 0.0126x – 0.0045	0.999	1.0 ± 0.8	1.7 ± 0.2	102.2 ± 0.8	101.8 ± 0.6

## Data Availability

Not applicable.

## Supplementary Materials

The following are available online at https://www.mdpi.com/article/10.3390/plants10102032/s1, Figures S1–S9: The 1H, 13C, COSY, HSQC, HMBC, and ROESY NMR, UV, IR, and HRMS spectra of compound 1, Figures S10–S18: The 1H, 13C, COSY, HSQC, HMBC, ROESY NMR, UV, IR, and HRMS spectra of compound 3, Figures S19–S20: The 1H and 13C NMR spectra of compound 11, Table S1: The quantitative analysis of the other 18 compounds.
