# Supplementary material for: Chemical Constituents of the Leaves of Diospyros kaki (Persimmon)
Source: Plants (Basel). 2021 Sep 28;10(10):2032. doi: 10.3390/plants10102032 (PMC8538319; doi:10.3390/plants10102032)
Supplement: Supplementary file 1 [file plants-10-02032-s001.zip › plants-1391408-supplementary.pdf]

plants

Article

# Chemical constituents of the leaves of *Diospyros kaki* (Persimmon)

**Jaeyoung Kwon [1,†], Jeong Eun Park [2,†], Jin Su Lee [2], Jung Hwan Lee [1], Hoseong Hwang [1], Sang Hoon Jung [1], Hak Cheol Kwon [1,*], Dae Sik Jang [2,*]**

[1] KIST Gangneung Institute of Natural Products, Korea Institute of Science and Technology (KIST), Gangneung 25451, Republic of Korea; kjy1207@kist.re.kr (J.K.); 217513@kist.re.kr (J.H.L.); hoseong91@kist.re.kr (H.H.); hkwon@kist.re.kr (H.C.K.)
[2] Department of Life and Nanopharmaceutical Sciences, Graduate School, Kyung Hee University, Seoul 02447, Republic of Korea; 530pje@naver.com (J.E.P.); lee2649318@naver.com (J.S.L.); dsjang@khu.ac.kr (D.S.J.)
* Correspondence: hkwon@kist.re.kr (H.C.K.); dsjang@khu.ac.kr (D.S.J); Tel.: +82-33-650-3504; +82-2-961-0719
† These authors contributed equally to this work and joint first authors

**List of Supporting Information**

**Figure S1.** The $^{1}H$ NMR spectrum of compound **1** (500 MHz, methanol-$d_4$).
**Figure S2.** The $^{13}C$ NMR spectrum of compound **1** (125 MHz, methanol-$d_4$).
**Figure S3.** The COSY NMR spectrum of compound **1** (methanol-$d_4$).
**Figure S4.** The HSQC NMR spectrum of compound **1** (methanol-$d_4$).
**Figure S5.** The HMBC NMR spectrum of compound **1** (methanol-$d_4$).
**Figure S6.** The ROESY NMR spectrum of compound **1** (methanol-$d_4$).
**Figure S7.** The UV spectrum of compound **1** (MeOH).
**Figure S8.** The IR spectrum of compound **1**.
**Figure S9.** The HRMS spectrum of compound **1**.
**Figure S10.** The $^{1}H$ NMR spectrum of compound **3** (500 MHz, methanol-$d_4$).
**Figure S11.** The $^{13}C$ NMR spectrum of compound **3** (125 MHz, methanol-$d_4$).
**Figure S12.** The COSY NMR spectrum of compound **3** (methanol-$d_4$).
**Figure S13.** The HSQC NMR spectrum of compound **3** (methanol-$d_4$).
**Figure S14.** The HMBC NMR spectrum of compound **3** (methanol-$d_4$).
**Figure S15.** The ROESY NMR spectrum of compound **3** (methanol-$d_4$).
**Figure S16.** The UV spectrum of compound **3** (MeOH).
**Figure S17.** The IR spectrum of compound **3**.
**Figure S18.** The HRMS spectrum of compound **3**.
**Figure S19.** The $^{1}H$ NMR spectrum of compound **11** (500 MHz, dimethyl sulfoxide-$d_6$).
**Figure S20.** The $^{13}C$ NMR spectrum of compound **11** (125 MHz, dimethyl sulfoxide-$d_6$).
**Table S1.** The quantitative analysis of other 18 compounds.

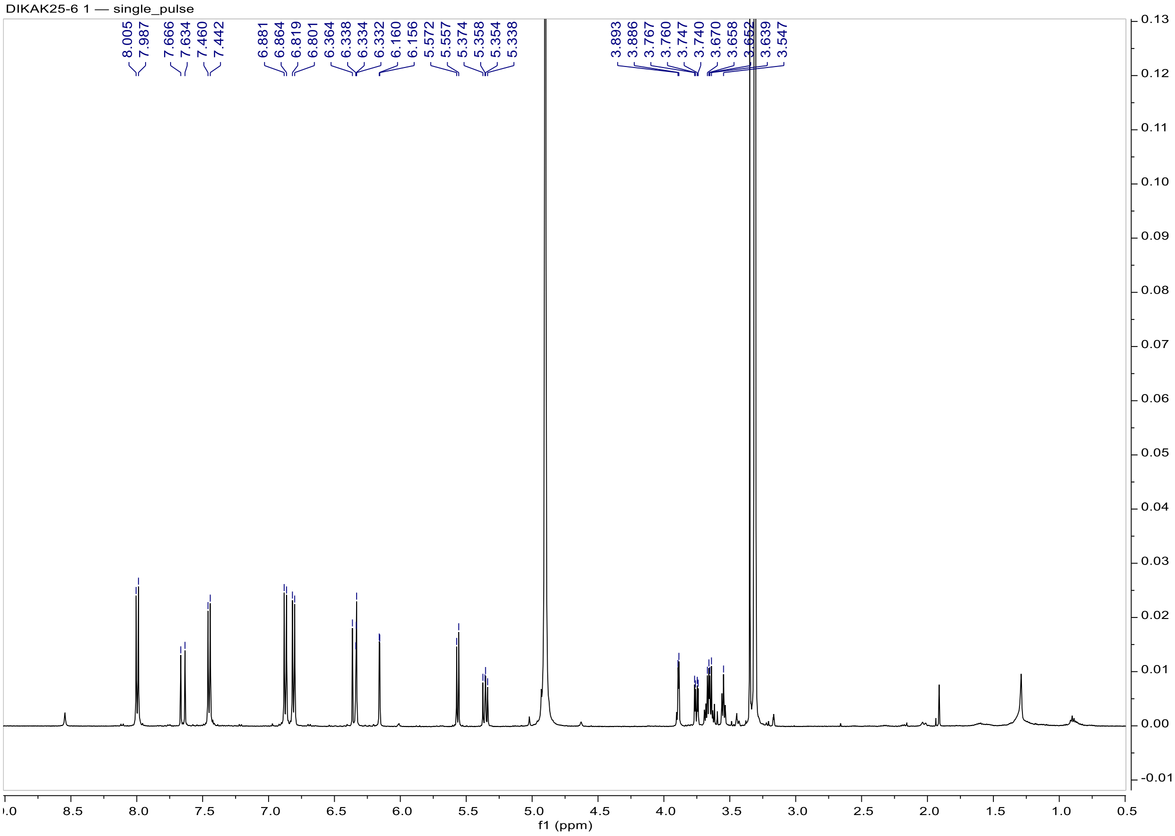

**Figure S1.** The $^{1}H$ NMR spectrum of compound **1** (500 MHz, methanol-$d_4$).

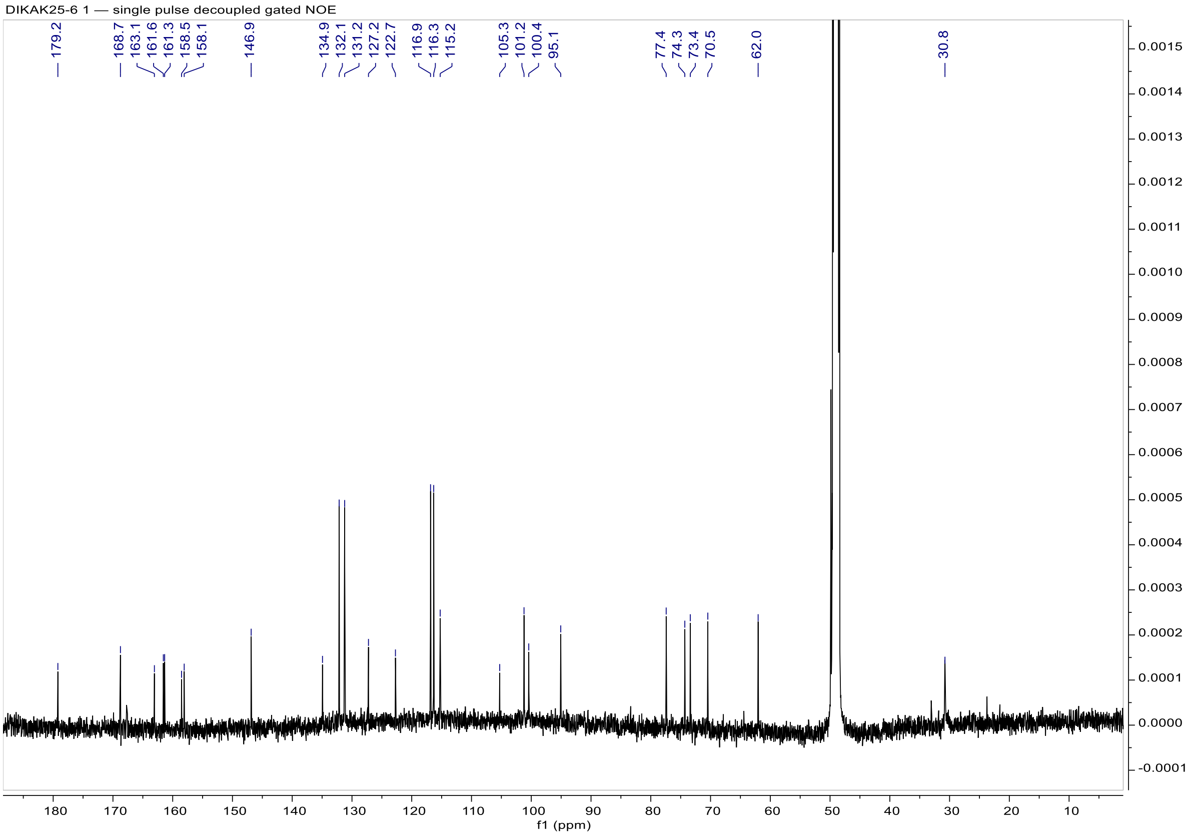


**Figure S2.** The $^{13}C$ NMR spectrum of compound **1** (125 MHz, methanol-$d_4$).

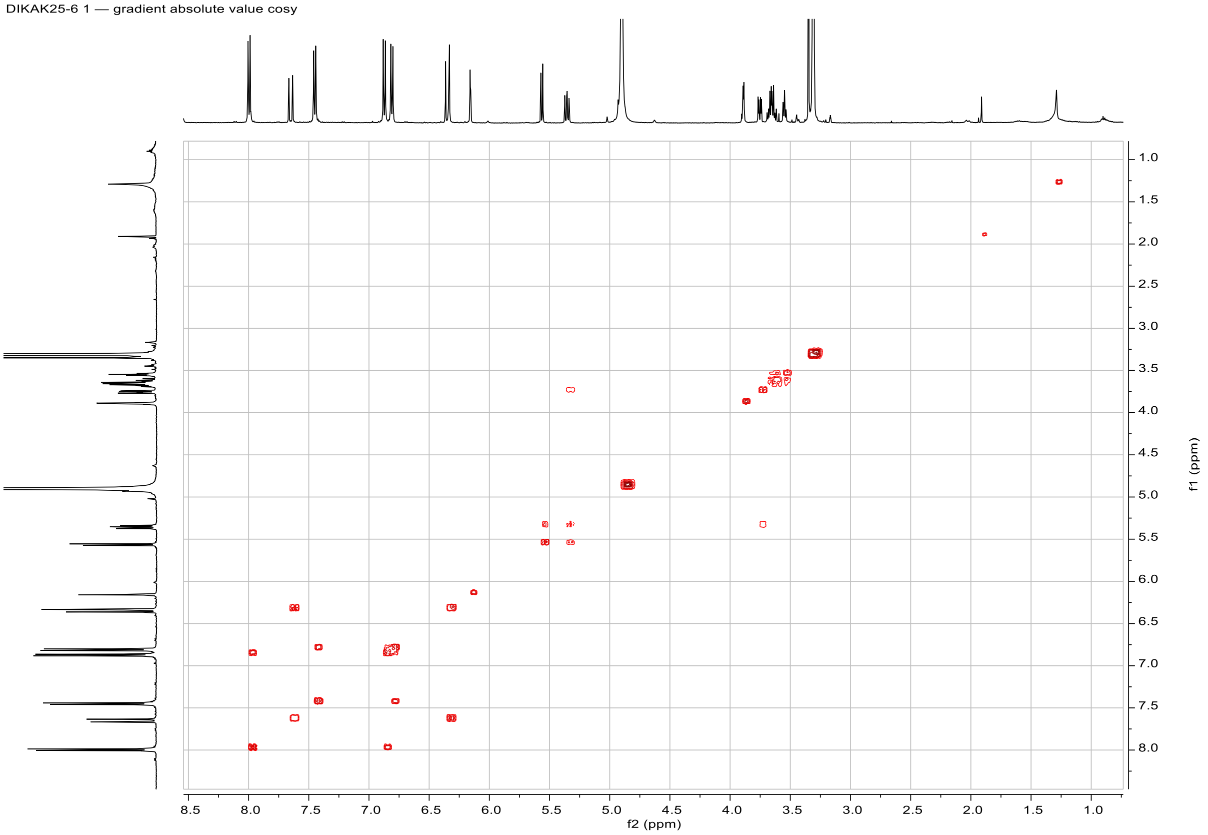


**Figure S3.** The COSY NMR spectrum of compound **1** (methanol-$d_4$).

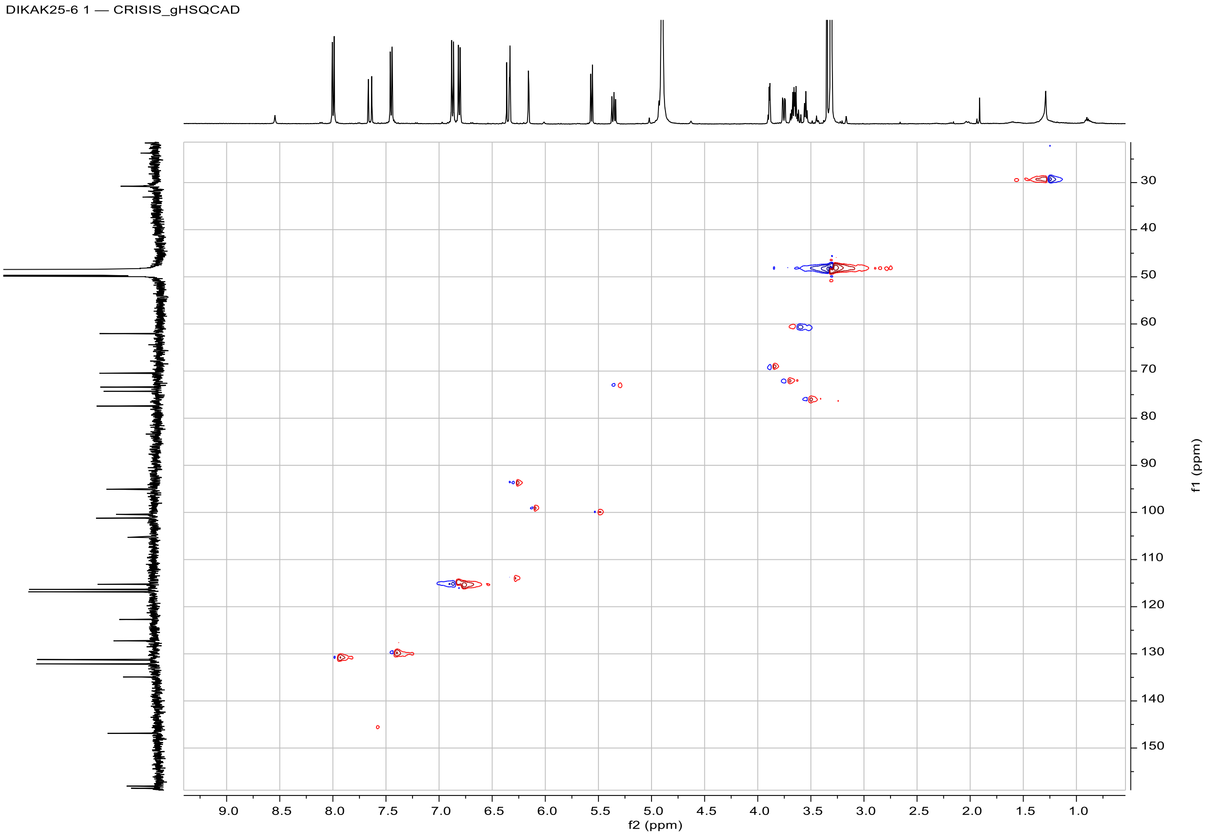


**Figure S4.** The HSQC NMR spectrum of compound **1** (methanol-$d_4$).

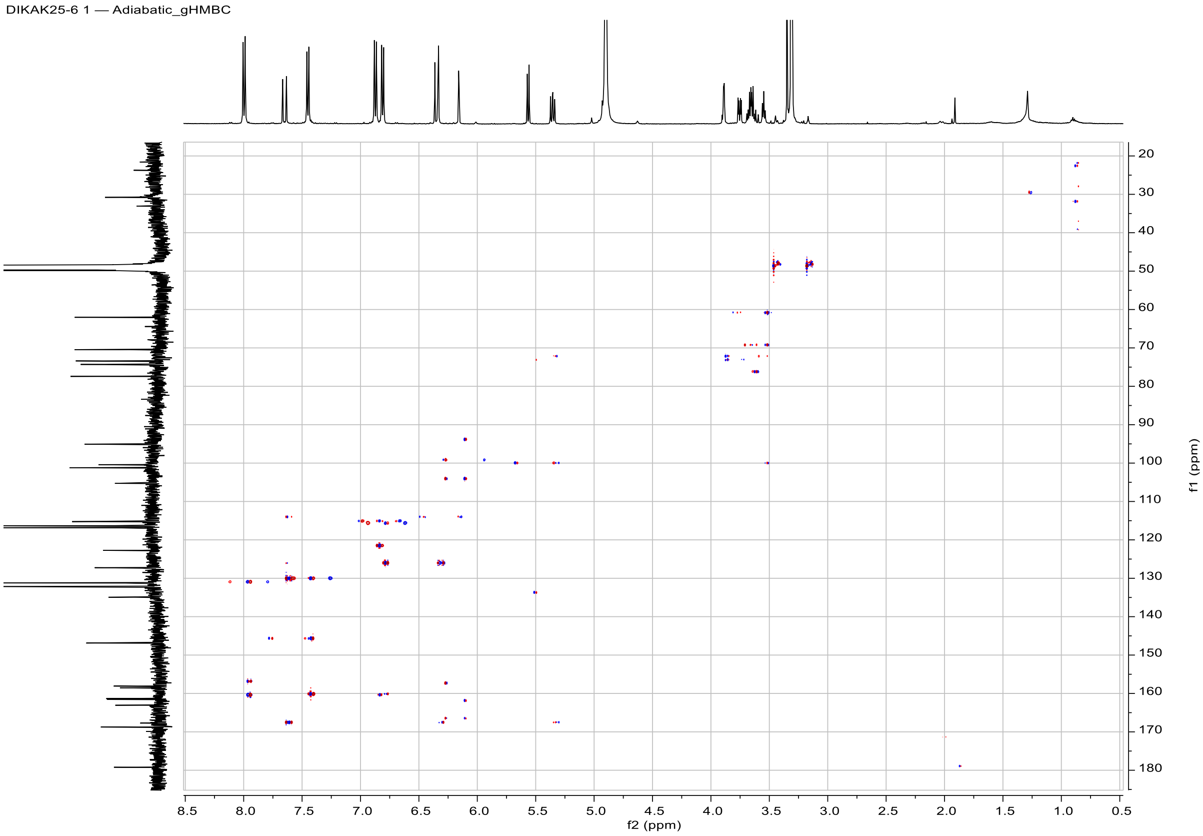


**Figure S5.** The HMBC NMR spectrum of compound **1** (methanol-$d_4$).

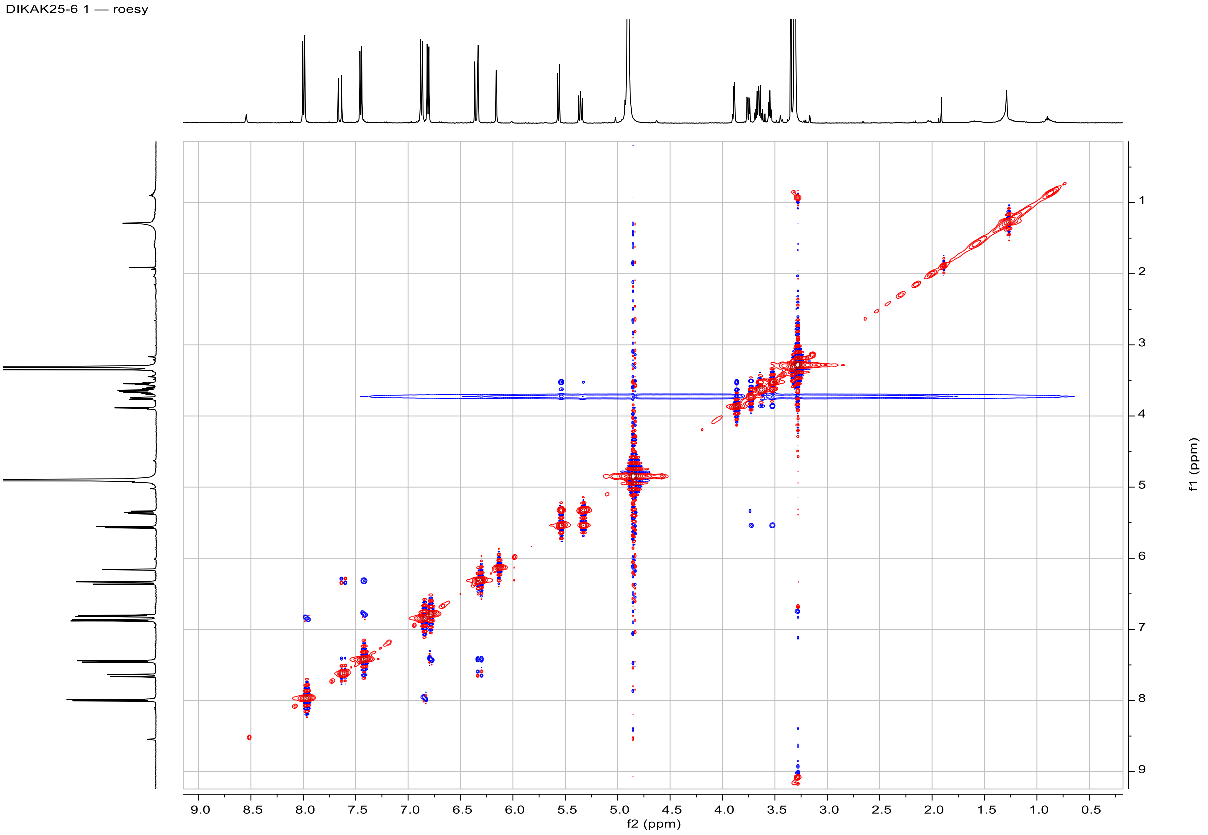


**Figure S6.** The ROESY NMR spectrum of compound **1** (methanol-$d_4$).

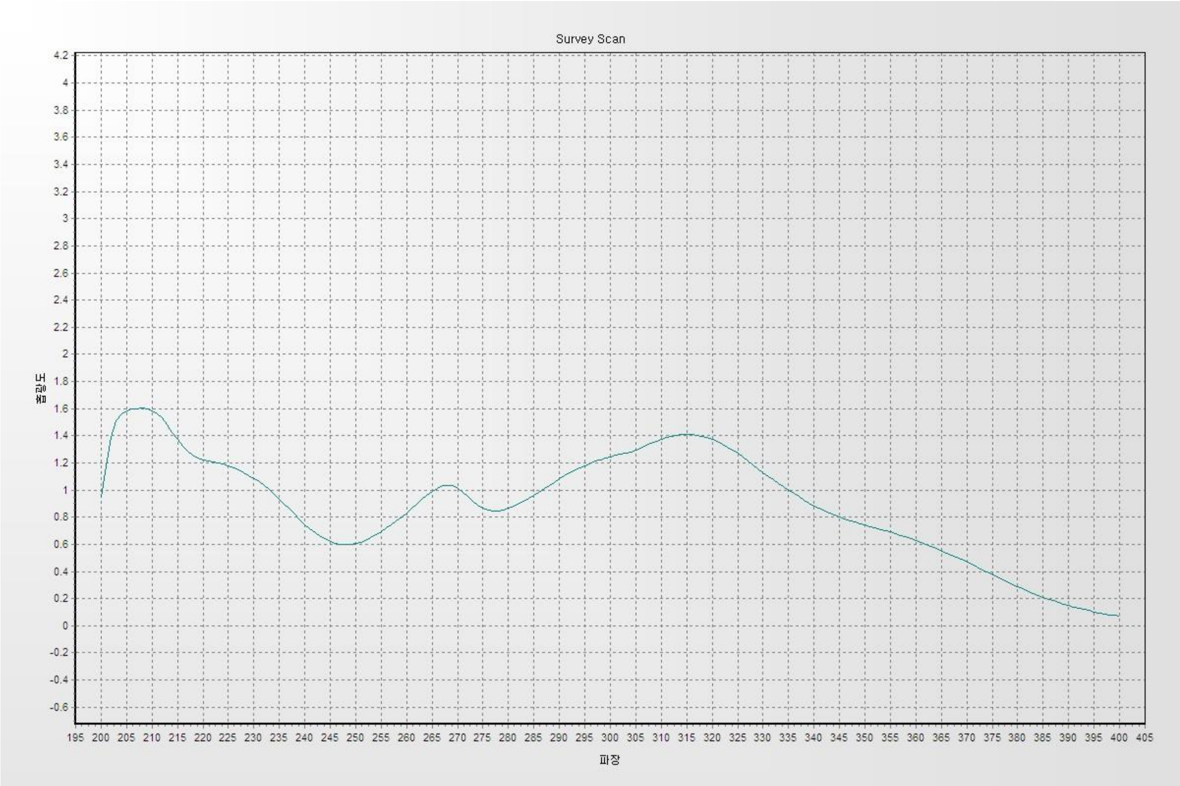


**Figure S7.** The UV spectrum of compound **1** (MeOH).

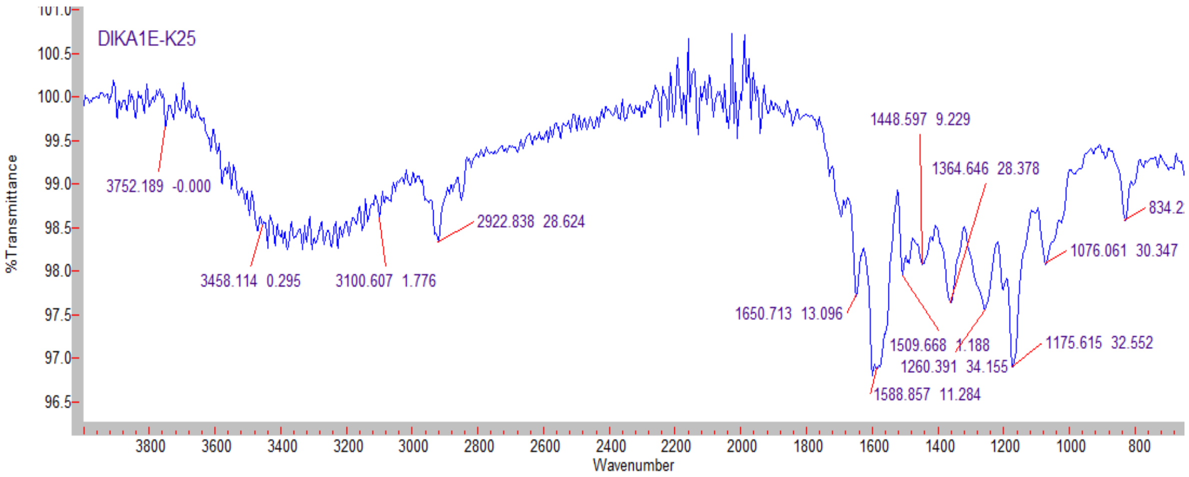


**Figure S8.** The IR spectrum of compound **1**.

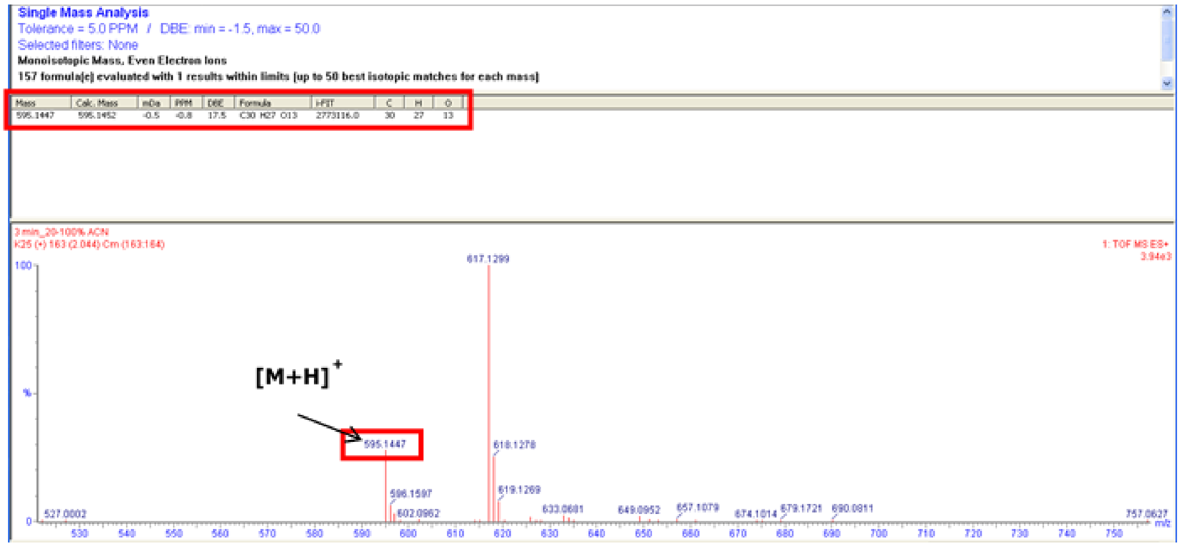


**Figure S9.** The HRMS spectrum of compound **1**.

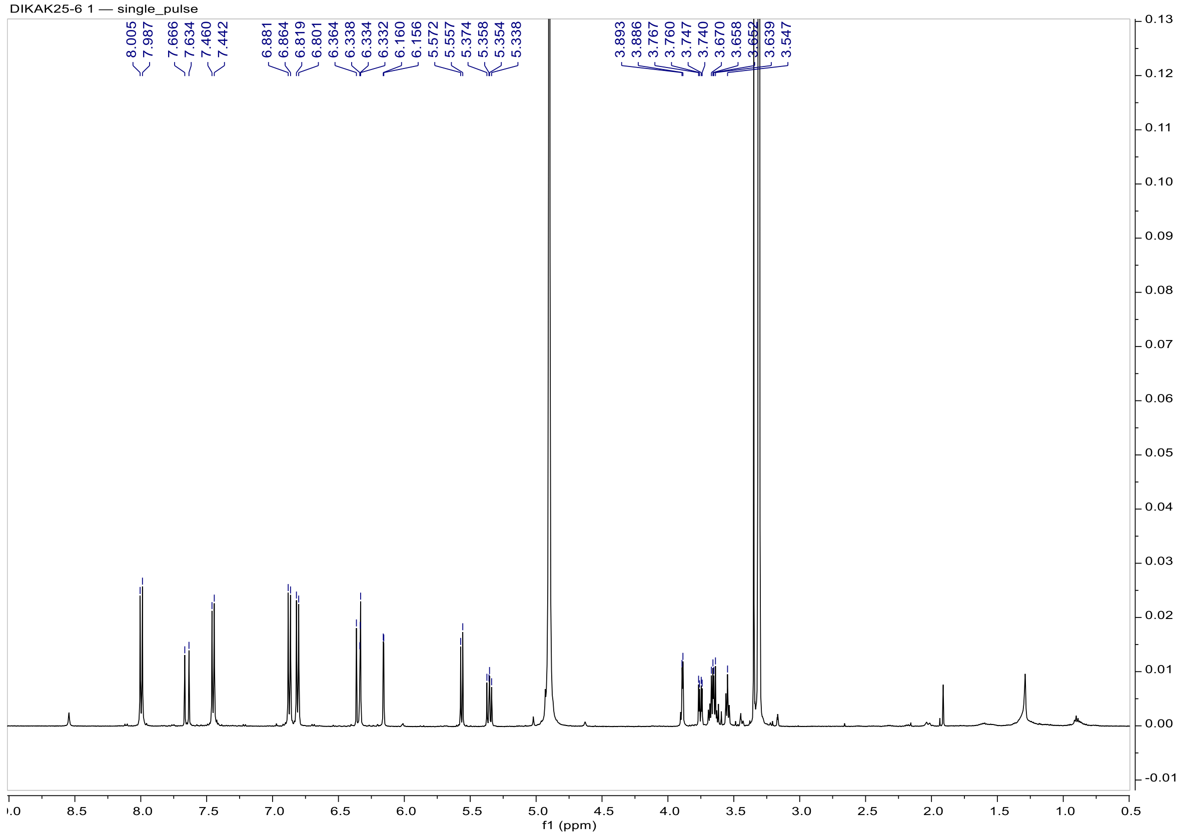


**Figure S10.** The $^{1}$H NMR spectrum of compound **3** (500 MHz, methanol-$d_4$).

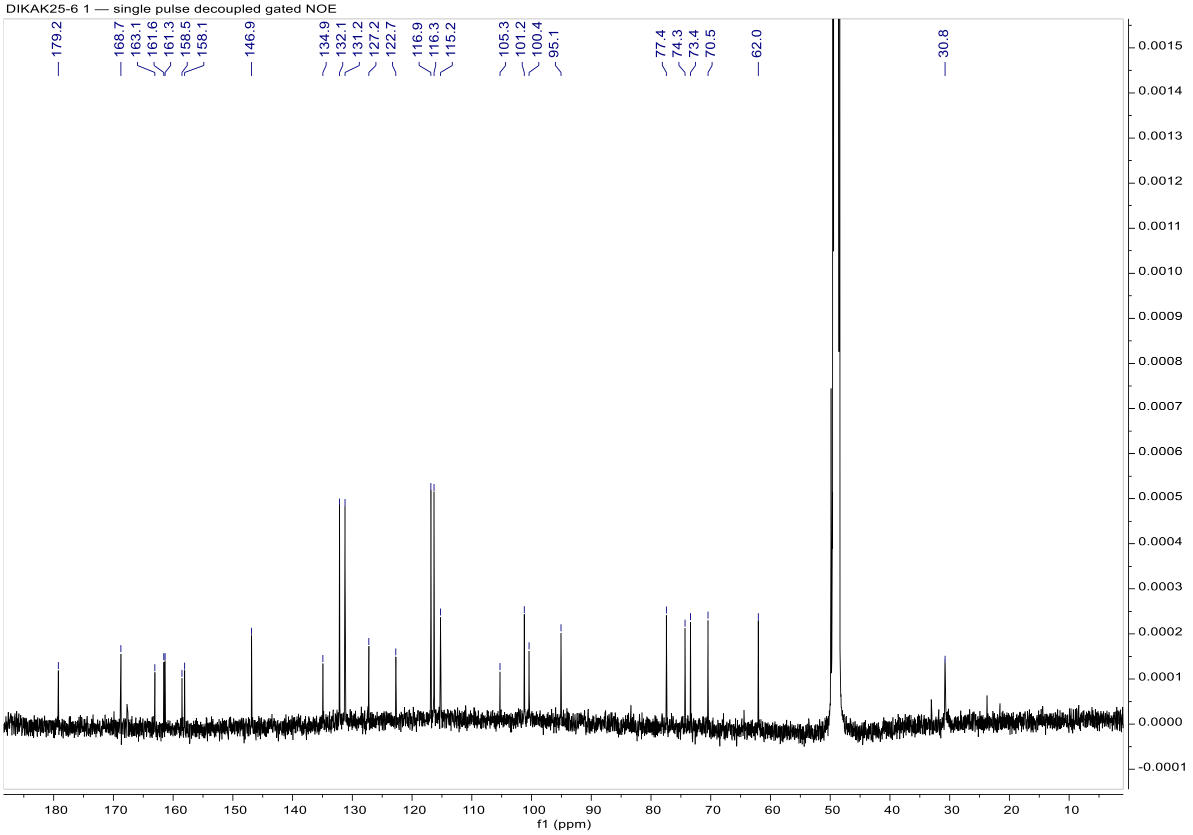


**Figure S11.** The $^{13}$C NMR spectrum of compound **3** (125 MHz, methanol-*d*4).

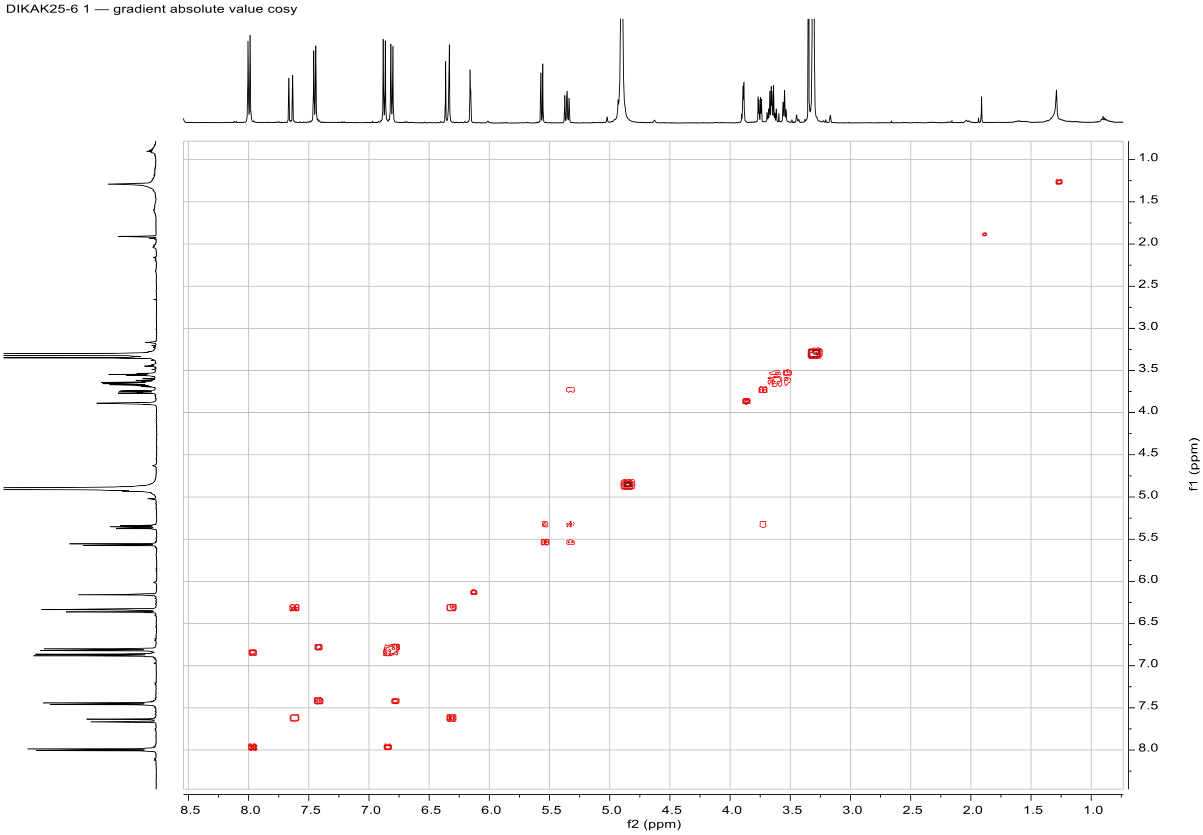


**Figure S12.** The COSY NMR spectrum of compound **3** (methanol-$d_4$).

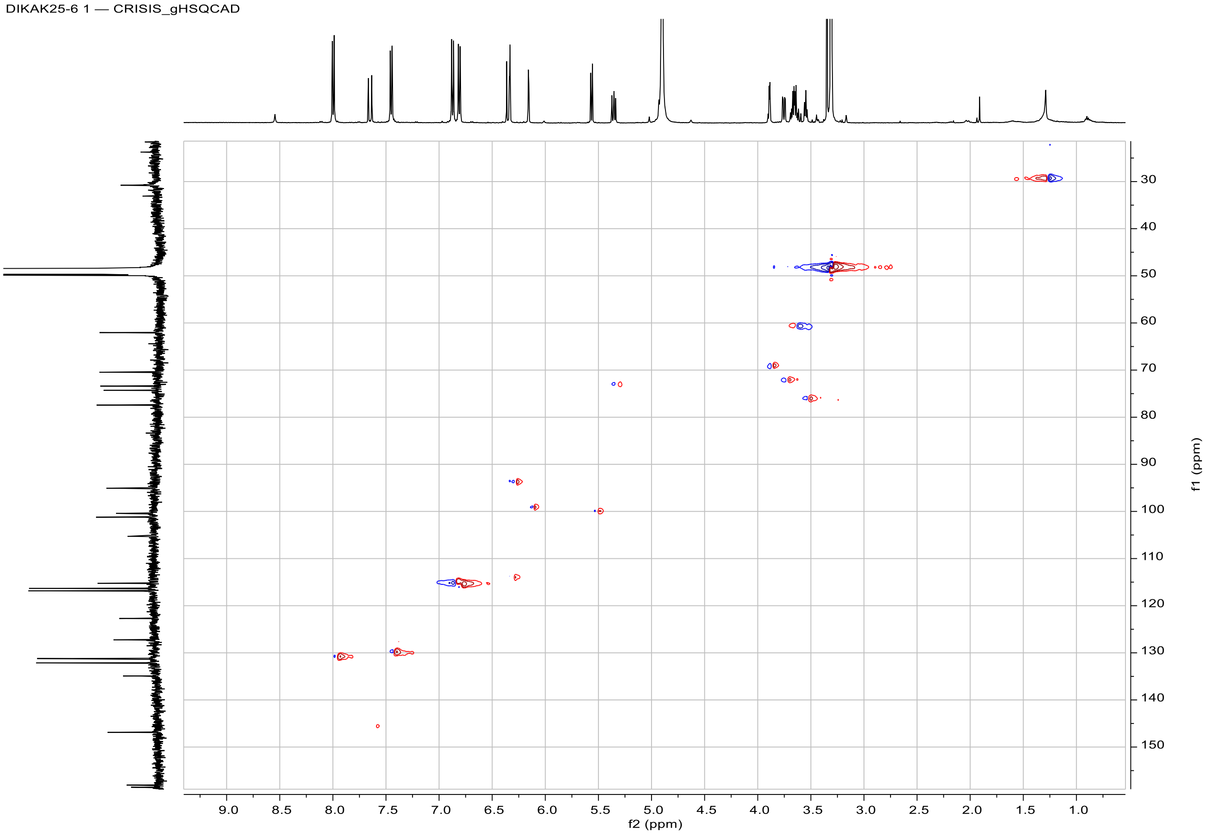


**Figure S13.** The HSQC NMR spectrum of compound **3** (methanol-*d*4).

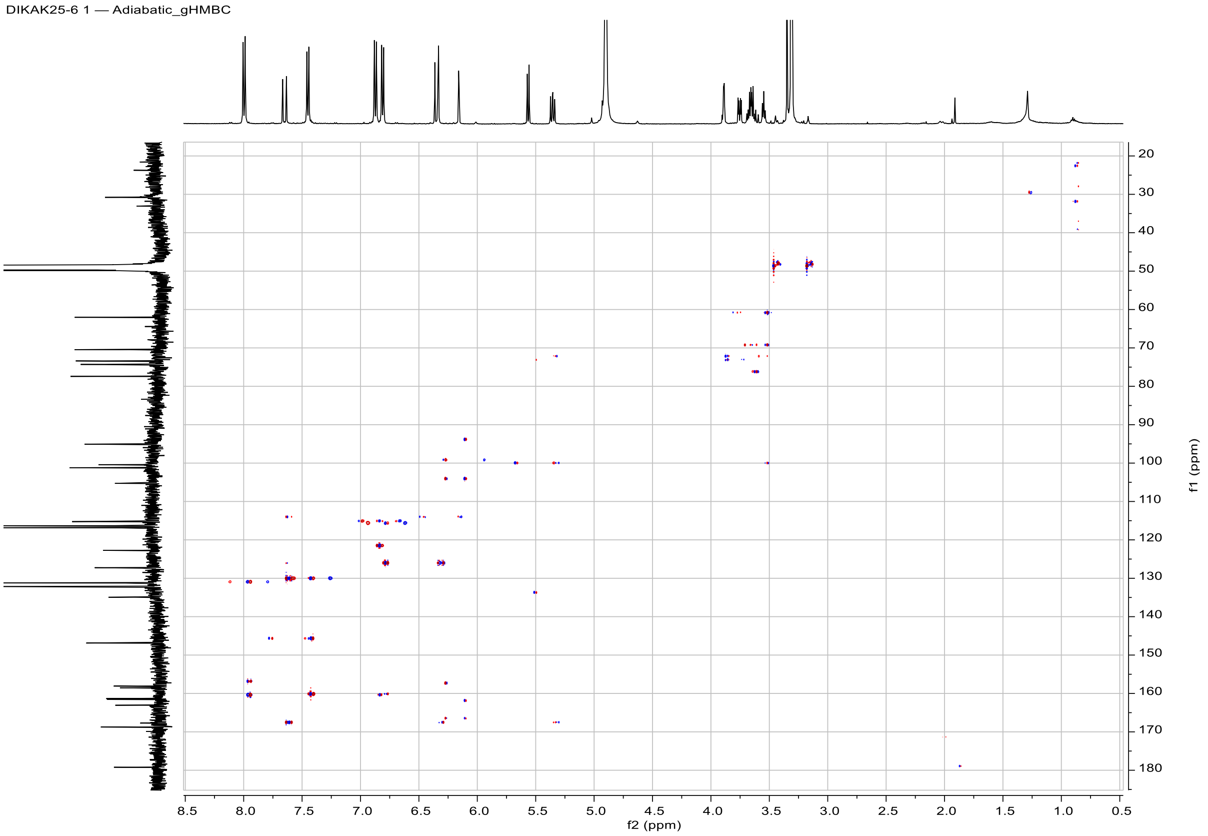


**Figure S14.** The HMBC NMR spectrum of compound **3** (methanol-$d_4$).

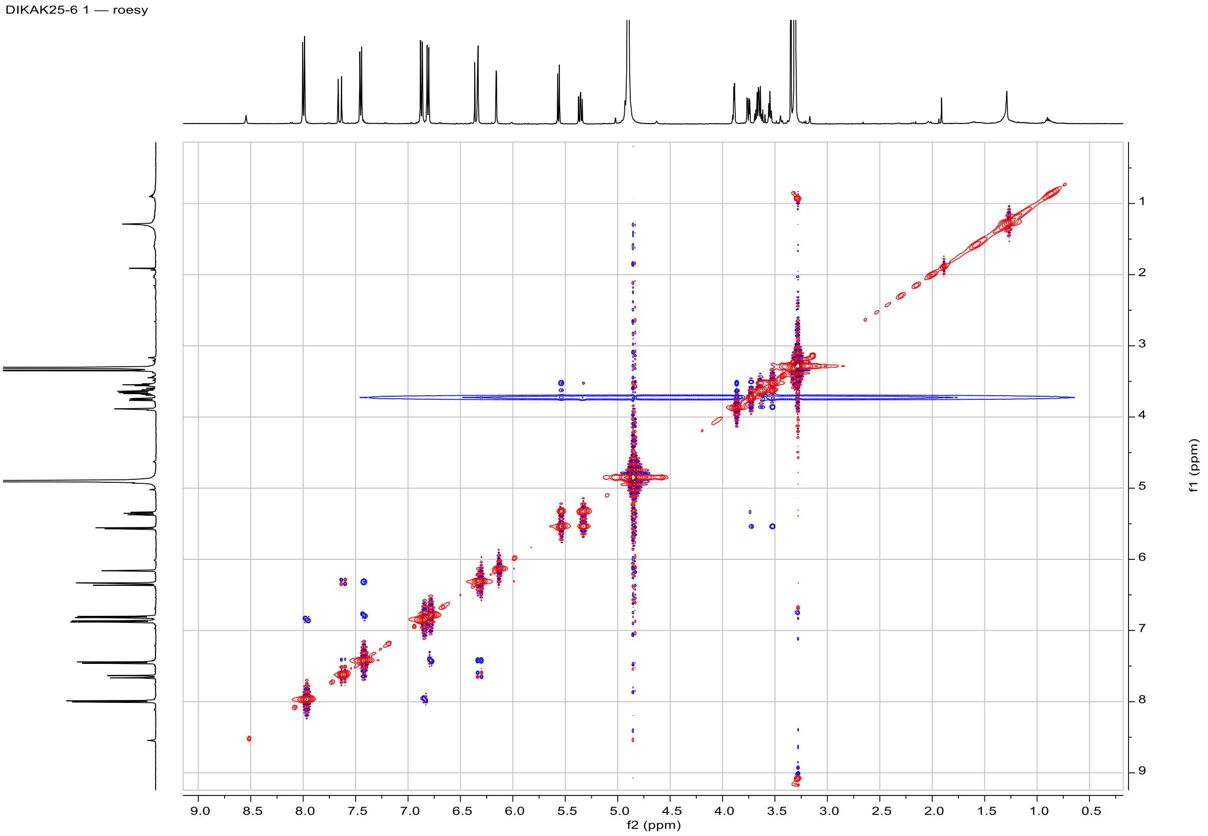


**Figure S15.** The ROESY NMR spectrum of compound **3** (methanol-*d*4).

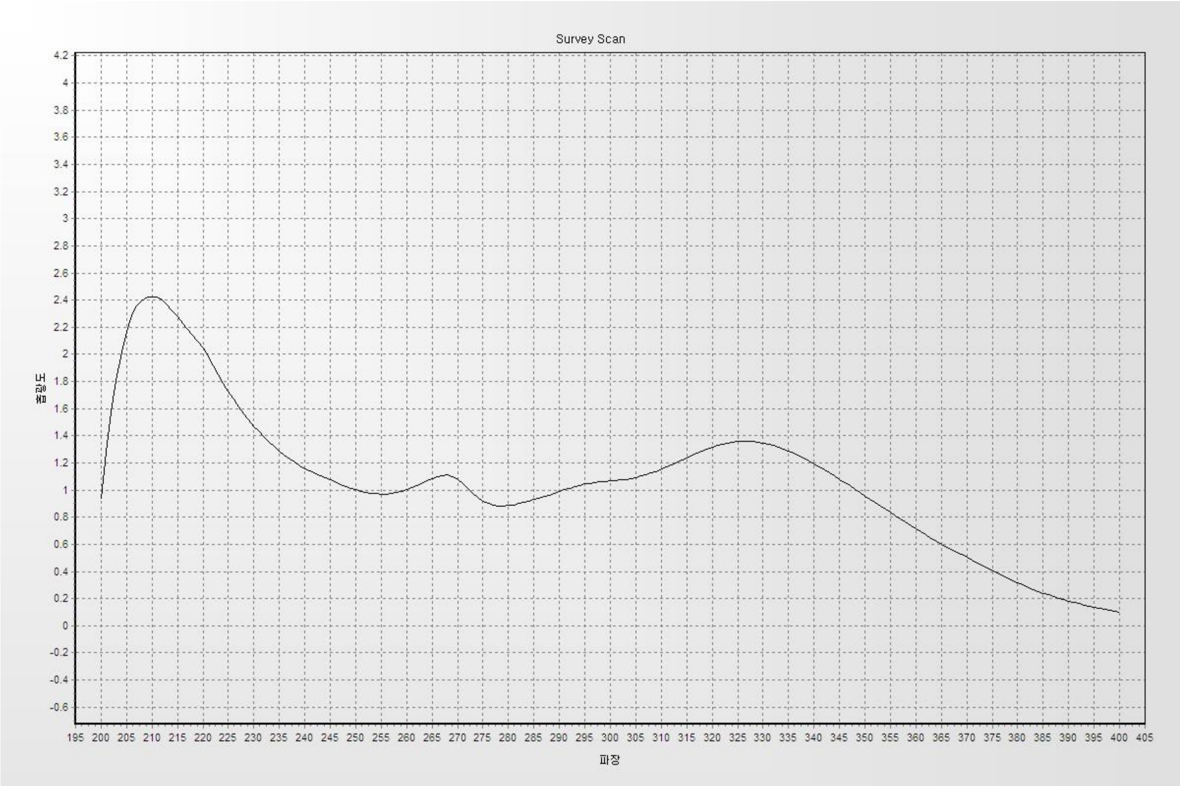


**Figure S16.** The UV spectrum of compound **3** (MeOH).

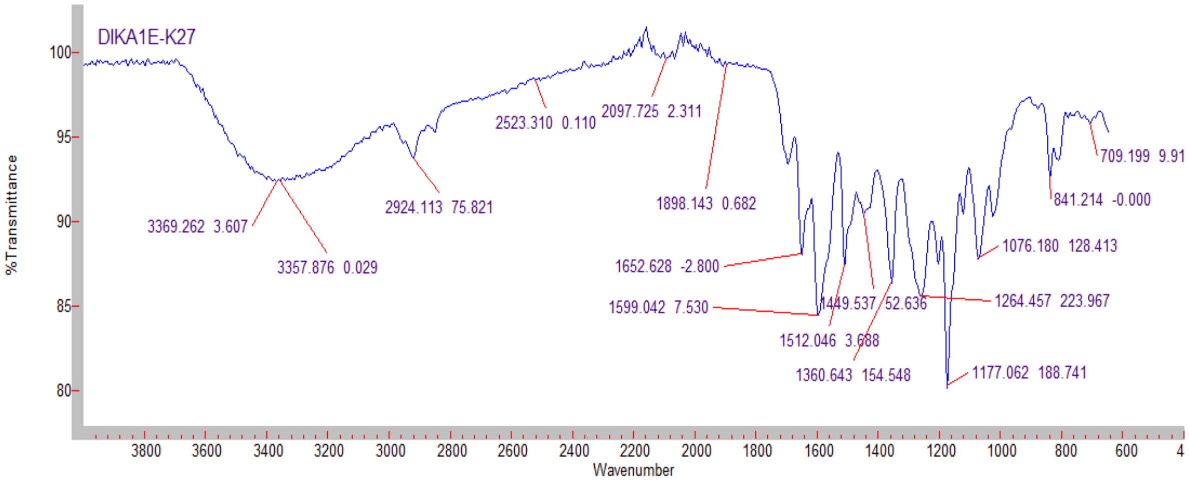


**Figure S17.** The IR spectrum of compound **3**.

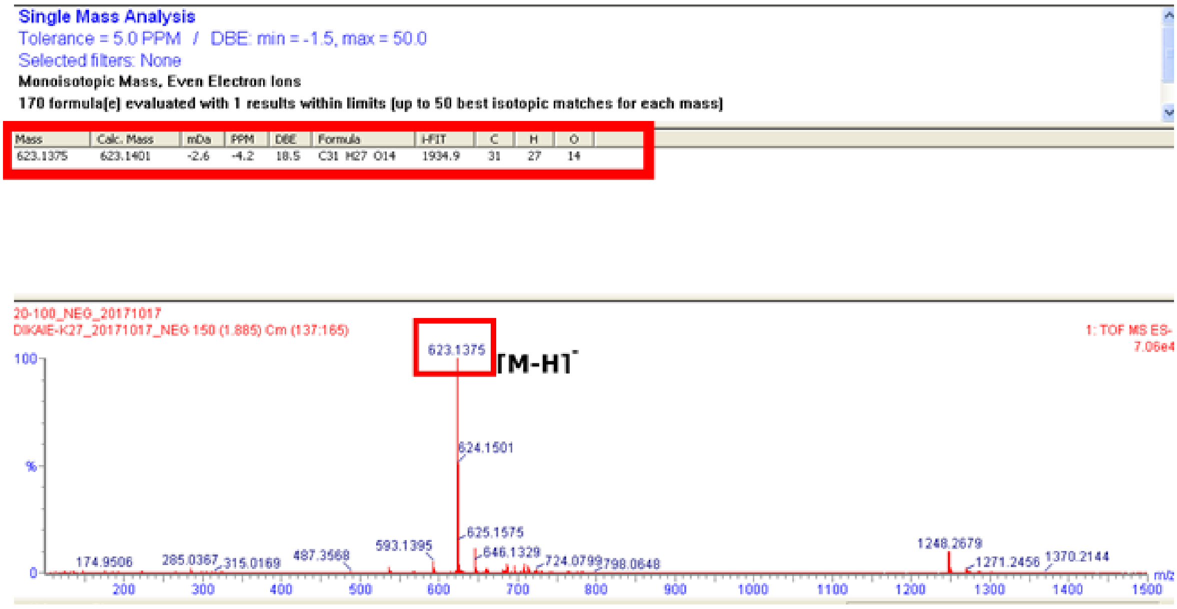


**Figure S18.** The HRMS spectrum of compound **3**.

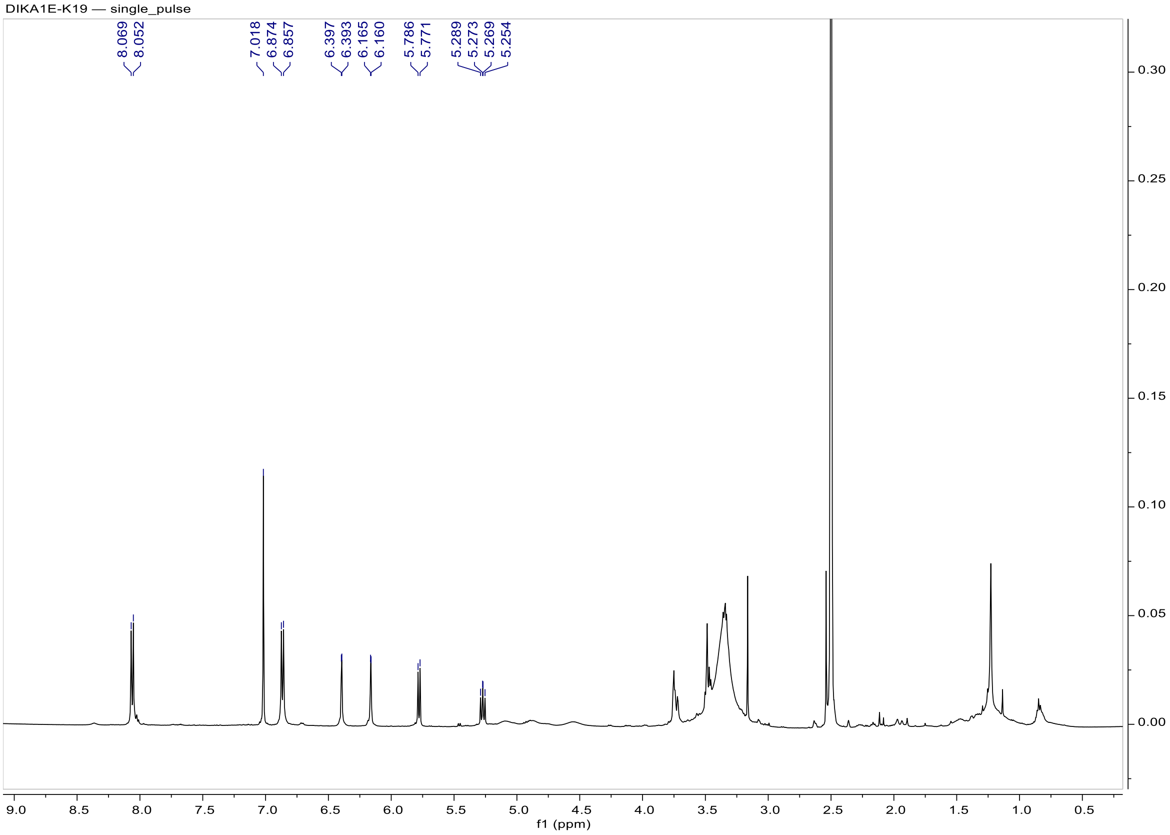


**Figure S19.** The $^{1}$H NMR spectrum of compound **11** (500 MHz, dimethyl sulfoxide-$d_6$).

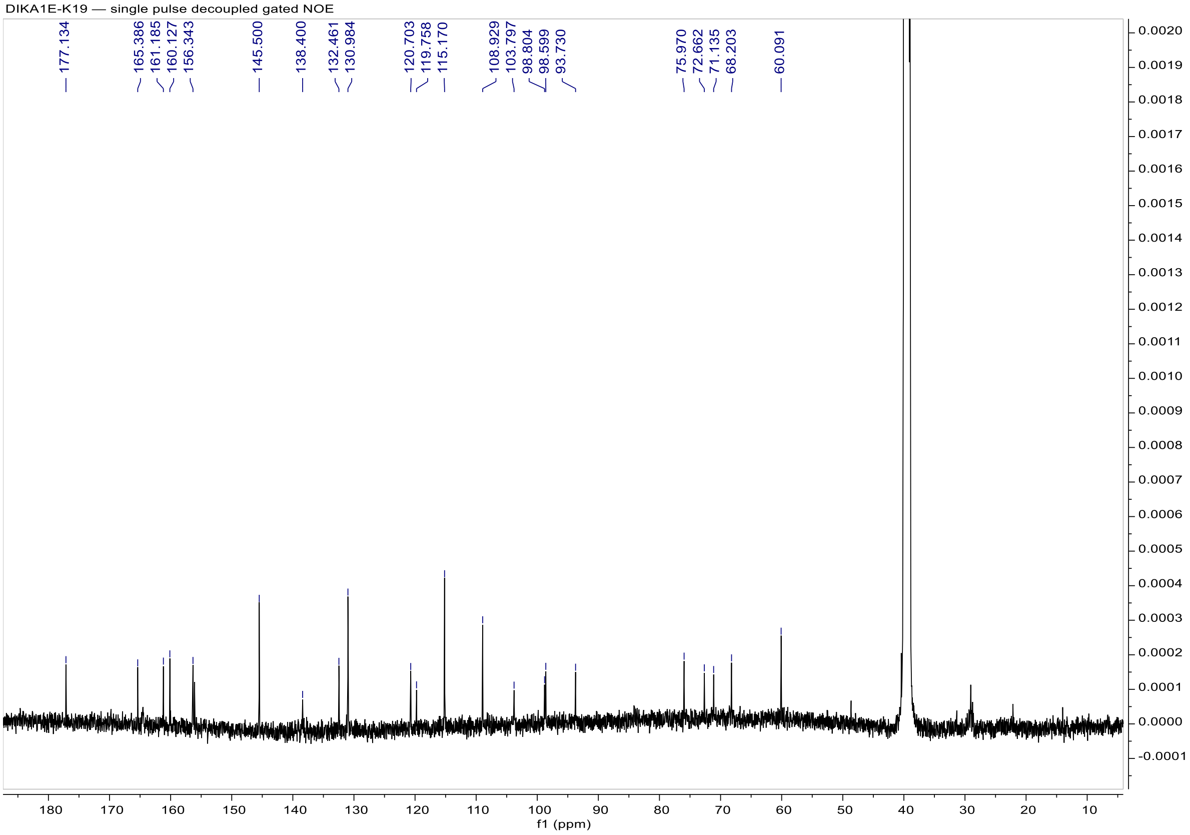


**Figure S20.** The $^{13}$C NMR spectrum of compound **11** (125 MHz, dimethyl sulfoxide-*d*6).

**Table S1.** The quantitative analysis of other 18 compounds.

| No | Content (%) | No | Content (%) |
|---|---|---|---|
| **1** | 0.01 | **19** | N/D |
| **2** | 0.01 | **20** | N/D |
| **3** | N/D | **21** | 0.89 |
| **9** | 0.30 | **22** | 0.38 |
| **10** | 0.72 | **23** | 0.59 |
| **12** | 0.10 | **24** | 1.25 |
| **14** | 0.01 | **25** | |
| **17** | 0.25 | **26** | 2.82 |
| **18** | N/D | **27** | |
